# Genetic/genomic testing: defining the parameters for ethical, legal and social implications (ELSI)

**DOI:** 10.1186/s12910-021-00720-5

**Published:** 2021-11-23

**Authors:** Tania Ascencio-Carbajal, Garbiñe Saruwatari-Zavala, Fernando Navarro-Garcia, Eugenio Frixione

**Affiliations:** 1 Program of Science, Technology and Society, Center for Research and Advanced Studies IPN (Cinvestav), 07360 Mexico City, Mexico; 2 Department of Cell Biology, Center for Research and Advanced Studies IPN (Cinvestav), 07360 Mexico City, Mexico; 3 Department of Legal, Ethical and Social Studies, National Institute of Genomic Medicine (Inmegen), 14610 Mexico City, Mexico

**Keywords:** Genetic testing, Genomic testing, Ethical, Legal and social implications, Bioethics, Genomic medicine, ELSI criteria, Genomic diseases, Patient rights, Policy-making, Healthcare

## Abstract

**Background:**

Genetic/genomic testing (GGT) are useful tools for improving health and preventing diseases. Still, since GGT deals with sensitive personal information that could significantly impact a patient’s life or that of their family, it becomes imperative to consider Ethical, Legal and Social Implications (ELSI). Thus, ELSI studies aim to identify and address concerns raised by genomic research that could affect individuals, their family, and society. However, there are quantitative and qualitative discrepancies in the literature to describe the elements that provide content to the ELSI studies and such problems may result in patient misinformation and harmful choices.

**Methods:**

We analyzed the major international documents published by international organizations to specify the parameters that define ELSI and the recognized criteria for GGT, which may prove useful for researchers, health professionals and policymakers. First, we defined the parameters of the ethical, legal and social fields in GGT to avoid ambiguities when using the acronym ELSI. Then, we selected nine documents from 44 relevant publications by international organizations related to genomic medicine.

**Results:**

We identified 29 ELSI sub-criteria concerning to GGT, which were organized and grouped within 10 minimum criteria: two from the ethical field, four from the legal field and four from the social field. An additional analysis of the number of appearances of these 29 sub-criteria in the analyzed documents allowed us to order them and to determine 7 priority criteria for starting to evaluate and propose national regulations for GGT.

**Conclusions:**

We propose that the ELSI criteria identified herein could serve as a starting point to formulate national regulation on personalized genomic medicine, ensuring consistency with international bioethical requirements.

## Background

Genetic/genomic testing (GGT) refers to complementary tools used for improving health and preventing diseases. Genetic testing detects specific mutations in the genome of a patient for identifying monogenic diseases. In contrast, genomic testing detects risk factors and predisposition to diseases involving more than one gene [1]. Thus, GGT can identify features in the DNA of a patient that may affect her/his health, helping physicians to (a) prevent or at least delay the appearance of resulting illnesses, (b) estimate disease risk for family members, and (c) avoid the risk of transmitting those risks to descendants [2, 3]. Since GGT deals with such sensitive personal information, which could significantly impact the life of a patient or their families, it becomes imperative taking into account ethical, legal, and social considerations when practicing it [4]. Key considerations are still not entirely well defined.

In order to address these issues, Ethical, Legal, and Social Implications (ELSI) studies were formally started in 1990 as part of the Human Genome Project, with an aim at identifying and confronting the troubles posed by genomic research that could affect individuals, their family members and eventually society at large. ELSI studies are today an interdisciplinary research area in constant evolution and expansion, currently embracing much more than intended at its beginnings 30 years ago. The complex connections among ethical, legal and social studies have resulted in the term ELSI being commonly understood as an integral set instead of an aggregate of independent elements, thus turning it into a somewhat fuzzy entity [5]. Additionally, there are quantitative and qualitative discrepancies in the literature when describing the elements that provide content to each field of ELSI studies regarding to genomic and genetic testing as well as public health on genomics and genetics [4, 6, 7]. These discrepancies are also notice in international documents published by international organizations. There is no clear distinction as to whether the elements of study addressed belong to the ethical, legal or social field; sometimes they are only cited as ELSI [8–10], or they are referred to only as ethical principles although they include the legal and social field [11, 12]. In addition, there are differences in how many and which ELSI criteria they belong [8, 13]. This lack of agreement in the elements that define ELSI, as well as in the criteria linked to the information that arises from the practice of GGT, can generate confusion in policy and decision makers, who may lose sight of the relevance or even the urgency of addressing certain issues, leading to difficulties in developing regulations with international equivalences regarding the use of genomic technology and hindering international scientific cooperation. These inaccurate policies and decisions may end up affecting the rights of patients (i.e. government decisions for implementing and expanding newborn screening programs that impact on children rights to health [14]). In some cases, autonomy may be affected, such as the case of the Havasupai Indian Tribe where the right to informed consent and to know or not the results of the tests was violated [15]. As well as lead patients to make harmful health choices (i.e. patients who do not seek prompt treatment due to a false negative result on direct-to-consumer testing (DTC) for the detection of the BRCA gene [16]. In addition, the lack of agreement on privacy issues may open the field to (a) leave personal genetic data of users unprotected, and therefore exposed to violation of their privacy and that of their families; and (b) misuse of the genomic information of a person by third parties with economic, health or discrimination consequences, among other hazards [6, 17, 18].

There have been significant efforts to address these issues [10, 19–22]. However, some of them have been biased towards the clinical research part, setting aside other essential ELSI criteria for GGT such as those of commercialization and health regulation criteria, making it difficult to define parameters to include particular aspects for GGT within the concepts of ELSI. Consequently, quantitative and qualitative inconsistencies remain in the official and research literatures, which hamper a uniform description of the elements that provide content to each ELSI study field and ELSI criteria. In this work, we focus on defining the ethical, legal and social implications (ELSI) for genetic/genomic testing (GGT), but to achieve this goal, we first specified the parameters that define the ELSI fields. This main goal was achieved through an analysis of the major international documents on genomic medicine published by international organizations. Our analysis and the data generated may be useful for researchers, healthcare professionals and policymakers as an unbiased, synthesized, comprehensive view of relevant ELSI topics.

## Methods

**Fig. 1:**
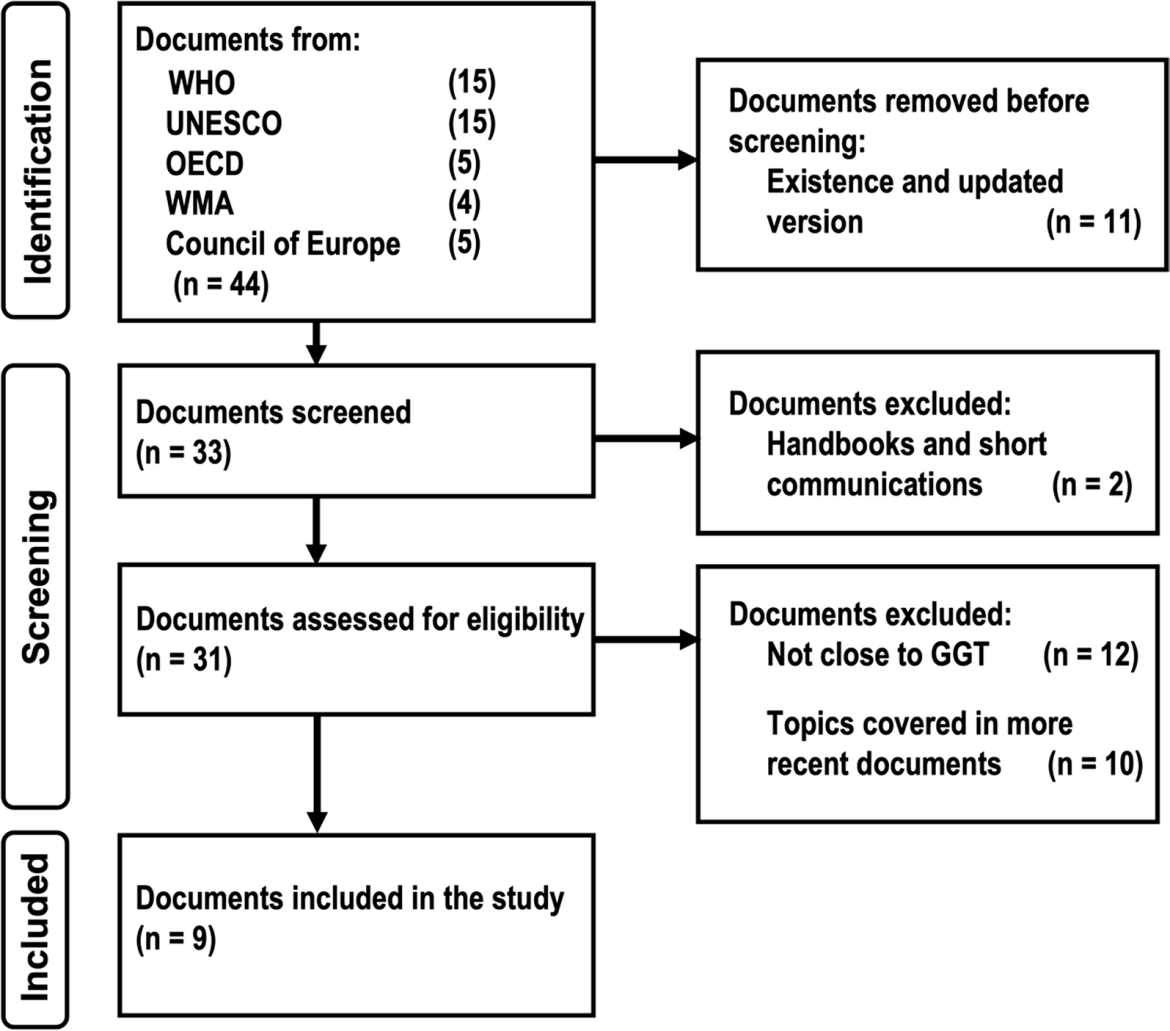
Flow diagram for selecting GGT-related content in databases of major international ELSI documents

In general, this study is limited to the ELSI aspects linked to GGT tests being carried out for disease detection or estimating the risk of developing one in adults with full consent capacity. In order to find out what exactly are the parameters that define ELSI criteria associated with GGT, we analyzed the documents published by international organizations related to ELSI subjects by following an adapted version of the steps recommended by Strech and Sofaer regarding a systematic review of reason [23], see also Fig. 1. This is because according to Boyle 1999, international documents are guides that help countries to regulate their own practices within, in this way soft law allows to create non-binding guidelines that becomes national binding regulations [24]. Additionally, in this work, we use the word "documents" in a sense that encompasses both instruments (binding for the signatory countries and non-binding) as well as recommendations made by panels of experts coordinated by international organizations. Thus, the international documents related to ELSI were subjected to the Strech and Sofaer systematic review based on four steps: (1) formulation of the review question and eligibility criteria, (2) selection of all the documents that applies to the criteria, (3) extraction and synthesis of information, (4) presenting the results with an answer to the review question.

The study question was defined as: What are the main ELSI criteria which international organizations have demanded for GGT over the last thirty years? Eligibility criteria were set as: documents published by international organization in the last 30 years, related to GGT tests for detection or estimating risk of diseases in adults with full consent capacity. To address the second step of the systematic review, we carried out a systematic search in databases of the major international organizations related to genomic medicine, mainly focusing on guidelines that could work for different countries rather than on particularities, according to Boyle (1999) as mentioned above. To identify the international documents concerning GGT, we carried out a systematic search of the terms “genomic medicine," "genetic testing" and "human rights and health" in databases of the major international organizations related to genomic medicine: World Health Organization (WHO), World Medical Association (WMA), Organization for Economic Co-operation and Development (OECD), United Nations Educational, Scientific and Cultural Organization (UNESCO), and Council of Europe. After several testing, these general key words were selected in order to minimize the exclusion of relevant documents, and for covering as much as possible by minimizing the bias that would leave some documents out. Next, the records of 30 years to date related to genomic medicine for health purposes in adults with full consent capacity were selected. Figure 1 shows the flow diagram for the identification of literature about ELSI of GGT and the result of it according to page et al. [25].

For the third step on extraction and synthesis of information, we carried out a systematic and detailed scrutiny of the identified documents. Each entire document was read to identify core concepts that met eligibility criteria in each article or statement. Final core concepts identified were reviewed, agreed and approved by all authors (differences were settled by majority): informed consent, non-discrimination, counseling, privacy and confidentiality issues, regulation, equity and accessibility, quality, trained medical personnel. A subsequent screening was performed to identify ideas associated with core concepts by either textual content or semantic ideas, and wrote it down as a "criterion" in a list. Finally, for the fourth step, these criteria were grouped by thematic affinity and thus defined as criteria and sub-criteria. Then these criteria and sub-criteria were assigned, by common agreement between authors, to the corresponding ETHICAL, LEGAL and SOCIAL field according to the definitions for fields indicated below.

Primary criteria in terms of coverage priority were then selected to be considered in regulations oriented to protecting all personal genetic information and rights. Finally, all data are discussed from present and future perspectives.

### Defining ELSI parameters

Defining the parameters for the ethical, legal and social fields is essential to avoid ambiguities when using the acronym ELSI. In order to address GGT criteria, first the ELSI fields were clarified. Although exist different definitions to differentiate ELSI fields [20, 26], we build along “ethical, legal and social issues in science and technology” guidelines proposed by Chameau et al. [27], because they clearly identify and separate ethical, legal and social fields. In addition, human rights themselves set guidelines on ethical principles for treating persons and therefore a minimum of universal standards for rights of patients [28]. Among universal ethical principles, those established by Beauchamp and Childress [29] (beneficence, non-maleficence, autonomy and justice) have resulted in a common framework for medical practice [30]. WHO has also developed guidelines for GGT services related to these principles [11], which were also used in this work to adapt bioethical principles to GGT.

Accordingly, ETHICAL criteria were defined as those based on the bioethical principles of beneficence, non-maleficence and autonomy [29], including those criteria that refer to respect for human rights and dignity. Within the LEGAL field we considered criteria that provide guidelines to regulate the activities of the parties involved in GGT, particularly those that entail authority, limits and procedures for decision-making—what is decided, who decides, and how is decided—, so as to guarantee rights protection of those involved. Finally, SOCIAL criteria were defined as those referring to the principle of justice, understood as distributive justice based on what would be desirable to achieve in an equitably just society. Therefore, we have included criteria focused on activities that allow access to genomic medicine services and communication, as well as dissemination of information to different spheres of society.

**Fig. 2:**
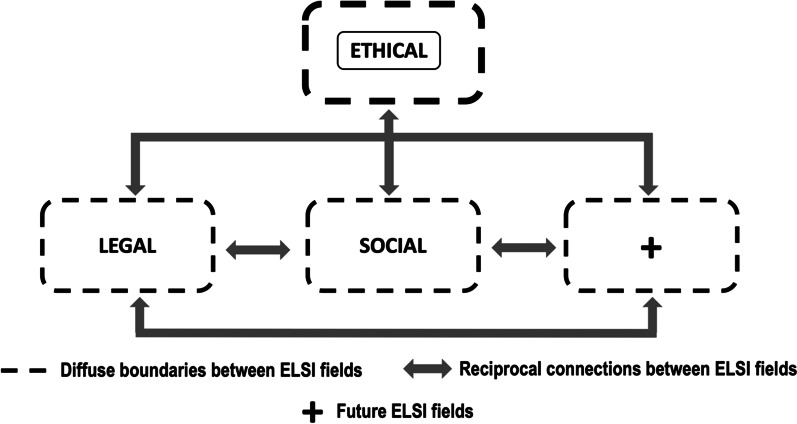
ELSI concept fields and their interconnections. The ETHICAL field is located at the top as mainstay for the rest of the fields—LEGAL, SOCIAL and future (+)—, which are placed in a lower hierarchical order. The interconnections between fields and the fuzzy limits among them are also represented

Thus, whole scheme with each field that makes up ELSI and the connections between fields, is illustrated synoptically in Fig. 2.

In conceptualizing the definitions of ELSI fields to build Fig. 2, we realized the ETHICAL field encompasses respect for human rights and prevails over all scientific or economic interests, acting as the pillar for the rest of the fields since it connects vertically and transversally with all of them; therefore, it is placed at the head of the scheme and enclosed within a continuous line box. After all, the main topic on GGT testing are based on universal principles inherent to the patient as a human being. Subsequently, we placed in a lower hierarchical order the legal and social fields, plus an unnamed field labeled with the " + " sign in anticipation of future inclusion of additional fields to the ELSI studies. All fields are delimited by dotted-line boxes to indicate fuzzy boundaries between them, as it is common for their topics of interest to overlap. Bidirectional arrows indicate reciprocal connections between all fields since there are always relationships between them, for a particular criterion usually covers two or more fields and can hardly be studied in isolation. As noted in Fig. 2 there is substantial synergy between all components of ELSI, though it was possible to disintegrate the whole scheme into its individual parts—ETHICAL, LEGAL and SOCIAL fields. This configuration allows giving specific content to each of them and setting their respective parameters, while permitting addition of future elements in an orderly manner, so it helped to proceed with the rest of the study in an easier and more organized way.

## Results and discussion

### ELSI criteria on genetic-genomic testing in bioethical international documents

A first general screening for the last 30-years period yielded a total of forty-four relevant sources, which once screened by recent publication dates and content close to GGT in terms of ethical, legal and social issues allowed us a final selection of nine documents for further detailed analysis (Table 1).

**Table 1:** International ELSI documents analyzed

Document	Agency	Year	Identification
Universal declaration on the human genome and human rights	United Nations Educational, Scientific and Cultural Organization (UNESCO)	1997	UDHG
Convention for the protection of human rights and dignity of the human being with regard to the application of biology and medicine: convention on human rights and biomedicine^a^	Council of Europe	1997	OC
Review of ethical issues in medical genetics	World Health Organization (WHO)	2003	REI
International declaration on human genetic data	United Nations Educational, Scientific and Cultural Organization (UNESCO)	2003	HGD
Declaration of Reykjavik—Ethical considerations regarding the use of genetics in health care	World Medical Association (WMA)	2019^b^	DR
Medical genetic services in developing countries. The ethical, legal, and social implications of genetic testing and screening	World Health Organization (WHO)	2006	MGS
Guidelines for quality assurance in molecular genetic testing	Organization for Economic Co-operation and Development (OECD)	2007	GQA
Additional protocol to the convention on human rights and biomedicine, concerning genetic testing for health purposes^c^	Council of Europe	2008	APOC
Report of the international bioethics committee on updating its reflection on the human genome and human rights	United Nations Educational, Scientific and Cultural Organization (UNESCO)	2015	IBC

^a^Better known as "Oviedo Convention". To date signed by 29 countries

^b^Adopted in October 2005 and subsequently revised in 2009 and 2019 by the WMA General Assembly

^c^To date signed by 6 countries

Other documents served as precedents since they also address bioethical issues related to human genetics, such as Nuremberg Code (1947) [31, 32], Helsinki Declaration (1964) [33], Asilomar Conference on the Risks of Recombinant DNA (1975) [34], Belmont Report (1978) [35], Declaration of Lisbon on the Rights of the Patient (1981) [36], Report of the Nuffield Council on the ethical issues of genetic screening (1993) [37], Genomics and World Health (2002) [19], Declaration on Bioethics and Human Rights (2005) [38], among others. These documents are important in their own fields and historical moments but were not included in this study because they are already considered in the main updated texts finally selected. Although these international documents include other branches of genomic medicine—like research, cloning, genetic editing, etc.—, we focus on the closest for addressing specifically the ELSI aspects of GGT.

We identified documents of two types, international instruments and international recommendations. International non-binding instruments, like UDHG (General Conference, 29th, 1997) and HGD (General Conference, 32nd, 2003), were approved by a majority at general conferences of UNESCO and therefore are not signed by member countries. For OC and APOC, they become binding instruments for signatories’ countries. Recommendations documents were prepared by international panels of experts and coordinated by the international organizations (documents REI, DR, MGS, GQA, IBC) thus they are not signed or approved by countries. Most selected documents show a supreme interest in the prevalence of ethical aspects, i.e., safeguarding human rights and dignity above any other economic, social, commercial or research interests (as we will demonstrate below in a further analysis). And, although they share specific criteria in relation to ELSI issues, there are differences on which of these should apply to GGT, as well as in their depth. The following particularities merit special attention in chronological order:

The UNESCO 1997 Universal Declaration on the Human Genome and Human Rights (UDHG) [39] is considered the cornerstone in the international legal framework of ethical principles for genomic medicine and has served as the basis for all subsequent documents on this matter. It emphasizes the supreme interest of human rights, dignity and fundamental freedom over any other interest.

The Oviedo Convention (OC) [40] held that same year is the first internationally binding document for the countries that sign up and ratify it. To date, only 29 countries have done so [41]. It expands the principles of the UDGH on which it is based, specifying criteria on research topics, “Informed consent,” and “Genetic counseling”, also promoting the use of genetic tests exclusively for health purposes.

The Review of Ethical Aspects in Genetic Medicine (REI) [11], published by WHO in 2003, is based—like the present study—on the four principles of bioethics proposed by Beauchamp and Childress in 1979 (autonomy, beneficence, non-maleficence and justice). It takes these into the context of the ethical principles involved in genetic health care services for different patient groups, and of the technological applications it addresses. Focusing on the medical provider-patient relationship, it delves into the desired characteristics of informed consent and advice from health personnel to the patient. This document sets public education as a critical factor in the development of genetic services, considering social factors such as the response of diagnosed people and the social attitudes in different groups, with descriptions of advantages, risks and circumstances recommended to perform GGT.

The International Declaration on Human Genetic Data (HGD) [42], published by UNESCO in 2003, establishes the principles for collecting, processing, using and storing human genetic and protein data, and the biological samples from which such data originate. Although it is aimed at medical and scientific research, its principles are extended also to other service areas of genomic medicine, including cross-data topics as well as their eventual destruction of biological samples, physical and electronical records of human genetic data. In addition, it establishes the need to create regulations and urges countries to work on them for regulating cross-border transfer of human genetic data, proteomic data and biological samples, in order to promote international medical and scientific cooperation and guarantee equitable access to such data.

The Declaration of Reykjavik (DR) on Genetics and Medicine [12], published by the World Medical Association originally in 2005 and updated by the end of 2019, covers the ethical aspects of medical practice in research and clinical practice. It approaches GGT from the perspective of responsibility of physicians in the previous and later stages of interpreting results, including a section on unexpected findings, elaborating on the contents of genetic counseling with details on the characteristics that preparation of health care professionals should comprise to assure a broad informed consent.

The 2006 WHO report on Medical Genetic Services in Developing Countries (MGS) [8] covers the ethical, legal and social implications of genetic testing and screening. It is a comprehensive document with a greater focus on the subjects of interest. It highlights the principle of distributive justice and includes social issues which block genetic medicine services in developing countries. It recognizes the importance of protecting the privacy and confidentiality of genetic data to avoid discrimination and stigmatization in society, underlying the importance and priority of education and open dialogue about genetic medicine for the benefit of both society and the patient. Hence, it encourages actions that facilitate decision-making, such as counseling and the creation of patient-support organizations. It further analyzes the safety and well-being of the patient through quality assurance in products and services, particularly by strengthening regulations on those related to genetic matters. Finally, recommendations are issued to improve the ELSI criteria for genomic medicine in developing countries.

The Guidelines for Quality Assurance in Molecular Genetic Testing (GQA) [43], published by the OECD in 2007, promote a minimum of international standards for ensuring the quality of practices in molecular genetic testing laboratories, through compliance with the international quality standards ISO 17025 [44] for laboratory accreditation, testing and calibration, as well as with the ISO 15189 [45] standards for medical laboratories. In addition, this document encourages international cooperation and increases confidence of society in the governance of molecular genetic testing, laboratory surveillance, traceability of results, quality in reporting of results, and addresses the issue of cross-border exchange of samples and information.

The 2008 Additional Protocol to the Oviedo Convention on Genetic Testing for Health Purposes (APOC) [22] delves deeper than the original into the ethical and social principles of genetic testing. It provides more detail on sample types, communication of risk to family members, non-directive genetic counseling, quality of services, consent for people who cannot do it themselves, and respect for the user's private life. Currently, only six countries have signed and ratified this additional protocol [46].

The most comprehensive publication so far is that of UNESCO International Bioethics Committee of 2015 (IBC). In its Reflections on Human Genome and Human Rights [21], the document identifies five main ethical principles and social challenges: (a) respect for autonomy and privacy; (b) justice and solidarity; (c) understanding of health and disease; (d) cultural, social and economic context of science; and (e) responsibility for future generations. It exposes select topics of recently developed applications of genomic medicine, such as direct-to-consumer testing and personalized and precision medicine, assigning responsibility to the parties involved: countries, researchers, academics, physicians, regulators, for-profit companies and media, addressing also concerns about distributive justice and international solidarity. This is the more informative document presently available on the bioethics of genomic medicine with an interdisciplinary outlook.

The nine documents above vary in terms of their overall perspectives and approaches, depending on the respective publishing organization or entity, but a few main themes are prevalent in some of them: (a) clinical care and doctor-patient relationship (OC, REI, DR); (b) protection of patient rights through quality services (HGD, GQA); (c) review of interdisciplinary ELSI issues (MGS, IBC). The UDHG and the OC published in 1997 responded to international concerns about the applications of scientific and technological advances in relation to human health. Both documents focus on the protection of human rights and dignity during clinical research, so they concentrate on the ETHICAL field of ELSI and only superficially touch on the SOCIAL and LEGAL fields. In contrast, the 2015 IBC document deals mostly on ELSI criteria for GGT, going beyond the ethical aspect of rights protection and into the legal and social aspects of the commercialization of GGT technologies. It covers the protection of rights for patients as required by the ethical aspect but proceeds to assessing explicit legal responsibilities by each of the participants. In exploring the social aspect, IBC encompasses activities to ensure vertical and horizontal flow of information about GGT, and the distributive justice condition of securing access to services and international collaboration. It is clear, therefore, that progressive development has occurred from the first documents in the late 1990s, concerned mainly with ethical aspects for protection of rights for patients in research involving human beings, to more recent documents where the topics of technology applications and commercialization of products and services have become steadily included from about 2020.

This same progressiveness, moving from the protection of human rights in research to concerns about the use and commercialization of technology, is observed with the emergence of transnational ELSI programs. The first programs to study ELSI aspects of the human genome, such as the National Human Genome Research Institute of the United States in 1990 [47], pursued ethical and legal aspects focused on research. In turn, the programs emerged later were directed to multidisciplinary topics such as (a) programs focused on supporting public policy development and decision making: the "GE3LS" program of the Government of Canada in 2000 [48], the “p3G2" program of McGill University in 2004 [49]; (b) oriented to international scientific cooperation for research in genomics and society: the program "ELSI2.0" in 2012 [50]; and finally (c) focused on data sharing: the program "GA4GH" in 2013 [51]. All of these programs denote the change in interest about human genome studies, from just individual and family rights protection up to exploring many other issues in technology application to human health, stressing the need of creating regulations that guarantee the protection of genetic data and promote international medical and scientific cooperation.

Since no document covers all ELSI criteria by itself, nor they provide similar coverage of GGT, it seemed appropriate to select several documents and analyze them in depth so as to appreciate the fullness of the ELSI criteria related to GGT, for finally integrating these into a single extract as follows below.

### Ethical, legal, and social criteria for genetic/genomic testing

As shown in Table 2, 29 ELSI sub-criteria related to GGT were identified, which could be then organized and grouped into ten basic criteria: two in the ETHICAL field, four in the LEGAL field, and four in the SOCIAL field.

**Table 2:** Ethical-legal-social criteria and sub-criteria for genetic/genomic testing

	Criteria	Sub-criteria	Reference
Ethical	Patient rights	Right to health	1, 3, 6, 8, 9
		Free and informed consent	1, 2, 3, 4, 5, 6, 7, 8, 9
		Knowing or not knowing results and implications	1, 2, 3, 4, 5, 7, 8
		Respect for privacy and confidentiality	1, 2, 3, 4, 5, 6, 7, 8, 9
		Respect for human dignity	1, 2, 4, 8, 9
	Non-discrimination	Avoid genetic reductionism	1, 2, 3, 4, 5, 8, 9
		Genetic exceptionalism	1, 2, 4, 6
		Avoid stigmatization	1, 3, 4, 5, 6, 7, 8, 9
Legal	Protection of the Information	Actions to ensure the protection of biological samples, and all physical and electronic information	3, 4, 5, 6, 7, 9
	Testing	Circumstances of application	2, 3, 5, 6, 7, 8, 9
		Advantages, disadvantages and limitations	1, 3, 4, 5, 6, 9
	Health regulation	Qualified health personnel	2, 3, 5, 6, 7, 8
		Surveillance	7, 8
		Medical-patient-company responsibility	3, 5, 7, 9
		Countries responsibility	1, 4, 6, 7, 9
		Analytical validity	4, 6, 7, 8, 9
		Validity and clinical utility	6, 7, 8, 9
		Laboratory accreditation	7, 8
	Commercialization	Direct-to-consumer testing	5, 6, 9
		Medical tourism	7
		Advertising	6, 7, 9
		Cross-border business	4, 7, 9
Social	Counseling	Pre-clinical and post-results	1, 2, 3, 5, 6, 7, 8, 9
		In clinic	1, 2, 3, 4, 5, 6, 7, 8, 9
	Training	Education and dissemination	1, 3, 4, 6, 7
	Reporting of Results	Concept of health and disease	3, 9
		Communication of the risks	3, 4, 5, 7, 8, 9
		Unexpected findings	3, 4, 5, 7, 8, 9
	Accessibility	Access to services under the principle of justice	1, 2, 3, 4, 6, 8, 9

1. Universal Declaration on the Human Genome and Human Rights, UNESCO 1997

2. Oviedo Convention, Council of Europe 1997

3. Review of Ethical Issues in Medical Genetics, WHO 2003

4. International Declaration on Human Genetic Data, UNESCO 2003

5. Declaration of Reykjavik, WMA 2019

6. Medical genetic services in Developing Countries. The Ethical, Legal, and Social Implications of Genetic Testing and Screening, WHO 2006

7. Guidelines for Quality Assurance in Molecular Genetic Testing, OECD 2007

8. Additional Protocol to the Convention on Human Rights and Biomedicine, concerning Genetic Testing for Health Purposes, Council of Europe 2008

9. Report of the International Bioethics Committee on Updating Its Reflection on the Human Genome and Human Rights, UNESCO 2015

The ETHICAL field was confirmed as the support for all the analyzed documents, since all three present fields note the importance of protecting human rights and dignity as prime ethical criteria, which in turn affect the legal and social spheres. Here are found criteria such as "Patient Rights" and "Non-discrimination," which correspond to the bioethical principles of beneficence, autonomy, and non-maleficence. The "Patient Rights" criterion groups together those related to undergoing a GGT test and are valid from the moment the patient arrives at a health institute, whether public or private, until the results are safeguarded indefinitely in the files of the responsible laboratories or destroyed, depending on the specific situation. The criterion of "Non-discrimination" refers to the patients right to be treated equally inside and outside the health institution, regardless of the results of their genetic/genomic analyses as stated in the Report of the International Bioethics Committee on the Principle of Non-discrimination and Non-stigmatization [52]. It comprises three sub-criteria: (a) “Avoidance of reductionism” on the overestimation of genetic influence and underestimation of behavioral, psychosocial and environmental factors; (b) “Genetic exceptionalism” refers to the special handling of genetic data given its nature of providing information on the current or future state of health the person, their family and may also have cultural significance; (c) “Avoid stigmatization” of a person because of a test result for him or her, the family, group or community. The “Non-discrimination” criterion is recognized as a fundamental right of patients and is the most referenced in the documents here analyzed, so we divided it into three sub-criteria (Table 2). The ethical aspect is present in all the analyzed documents, since the ELSI studies began by covering the ethical field and therefore it has been examined for a longer time. Both "Patient Rights" and "Non-discrimination" are criteria that must always be accomplished and include mechanisms to be put into effect throughout the testing process. They represent a cross-cutting field through all other aspects of ELSI.

**Fig. 3:**
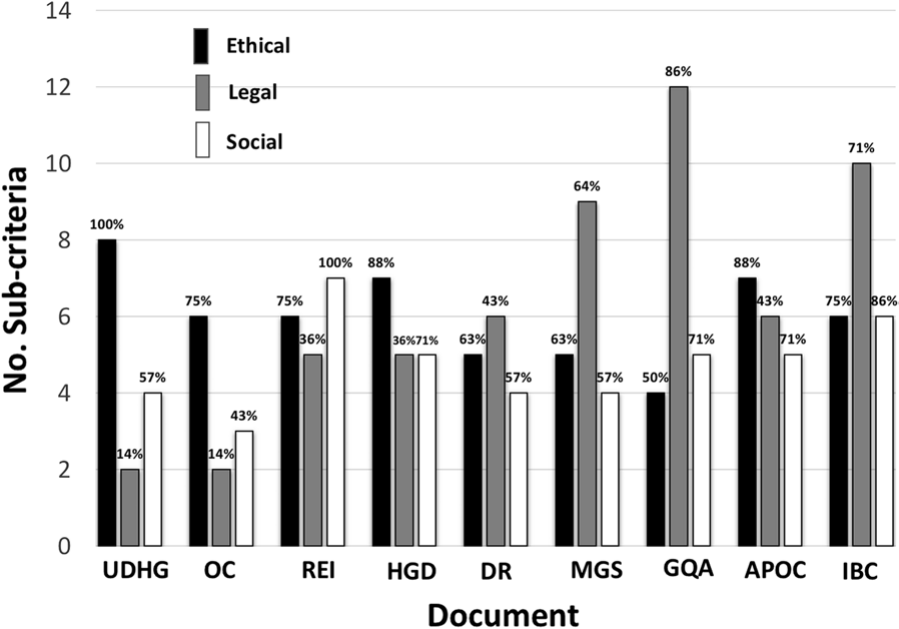
Number of sub-criteria covered by each international document divided into ETHICAL, LEGAL and SOCIAL fields. The percentages indicate how much of the total identified sub-criteria is covered by each field

In fact, an analysis of each of nine documents that denotes the number of ethical, legal, or social sub-criteria covered and the respective percentage for field, showed that most of documents are oriented into the ethical field (Fig. 3). Thus, the highest percentages were observed in following documents: UDHG 100%, OC 75%, HGD 88%, DR 63%, APOC 88% and MGS 63% (which practically tied to the social field, 64%). While REI and IBC documents have a greater number of criteria covered in the social field, and only GQA stands out in the legal field, of course related to quality and regulation issues. These data support the assumption that some criteria might be incomplete in a single document and therefore the need to complement each other to build a more complete guideline.

The LEGAL field contains most of the criteria and sub-criteria (fourteen) involved in GGT (Table 2), but they are not equally represented among the documents. Some of these, like the UDHG and OC, include only few legal sub-criteria like "Testing" and "Health Regulation"; furthermore, these documents do not delve into them. The "Protection of the Information" criterion focuses on time and form mechanisms to ensure safeguarding of biological samples, as well as any physical or electronic access to genetic information of the patient by unauthorized third parties, including destruction of both samples and data. The "Testing" criterion specifies under what circumstances it is advisable to perform a GGT test on the patient or a relative, informing the scope and limitations of the test. The "Health Regulation" criterion is the most extensive in content. It groups together the operations in which the legal regulatory and justice apparatus of each country must intervene to ensure the quality and reliability of GGT tests. The "Commercialization" criterion widens this scope by including sub-criteria related to the provision of GGT services and products, up to integrating technology commercialization with ELSI considerations, as it is found in the GQA and IBC documents. Yet this criterion was the least approached, denoting the research focus of most of the documents and reflecting also the international scene, where there is still no total convergence in a specific regulatory legal path for GGT, a pending situation for which the present study might be beneficial. It will be up to each country adopting proper regulations according to its particular circumstances, considering the international standards mentioned in Table 2 and always taking into account the cross-cutting ethical aspects.

As regards the SOCIAL field, we found criteria that refer to the bioethical principle of distributive justice, as well as criteria for accessing and communicating genetic information. Thus, four criteria are shown in Table 2. "Genetic Counseling" describes the characteristics of such advice—non-directive, complete, with simple language, respectful—, which health professionals must carry out at the different stages of testing until solving all the doubts the patients might have. The "Training" criterion includes public policies in genomics, training of qualified human resources in this area, dissemination of related activities to different spheres of society, wide availability and accessibility of information on genetic testing through country institutions, as well as by academic and civil organizations. The "Reporting of Results" criterion as part of the social field, covers processes by which healthcare professionals communicate test results, associated information and address unexpected findings to the patients and in some cases to their families, this helps to mark the boundary to avoid genetic determinism. This means avoiding the consideration of a GGT result as a disease when this has not appeared, and ruling out other environmental, biological or psychosocial factors. Finally, the "Accessibility" criterion is intended to highlight the vital importance of finding ways by which GGT tests are made available to the entire population that could require them for health purposes, and not exclusively to those who can afford them. In fact, GGT accompanied by adequate medical counseling can serve as a routine instrument of public health care for opportune disease detection and prevention, and for reducing social inequality in this regard. Therefore, the "Genetic Counseling" criterion in its different phases of testing is the one that stands out the most. The other three SOCIAL criteria are well represented in the documents because of their importance, although general implementation guidelines for putting all of them into effect in countries are still lacking.

### Priority criteria for genetic/genomic testing

It is also of vital importance to determine the priority criteria for genetic testing. Despite the relevance of each criterion here identified for GGT, not all of them are perceived with the same level of priority by experts in the international community. Criteria prioritization in the documents can be divided into four different groups. (A) Criteria that prioritize the safeguarding of dignity and human rights, (B) priority criteria to provide quality services regarding the protection of the health and the best interests for the patient, (C) prioritization approach to promote fair access to technology and health, (D) prioritization approach of the doctor-patient relationship.

**Fig. 4:**
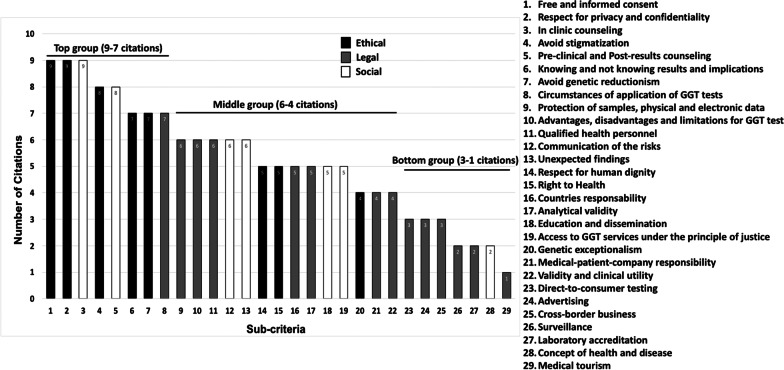
GGT Sub-criteria arranged according to the number of documents in which they appear

As mentioned above, the scrutiny of the nine documents here analyzed shows differences in the attention given to the criteria and sub-criteria they contemplate. Figure 4 presents the coverage of the 29 sub-criteria ordered by numbers of citations in the analyzed documents.

The numbers of citations for sub-criteria may be divided into three groups (Fig. 4). The top group encompasses seven to nine citations (sub-criteria 1 to 8), including almost entirely those comprised in the two ETHICAL field criteria of "Patient Rights" and "Non-discrimination". This is expected, as the rights of patients constitute the basis for all ELSI documents referring to the human genome. Only one sub-criterion in this group belongs to the LEGAL field, for it concerns when it is valid applying a GGT test. This sub-criterion is highly referenced since these tests are recommended only for health purposes and under medical recommendation, seeking to discourage excessive use of GGT technology for mere curiosity (ancestry) or without proper medical indication, which could lead patients to making harmful decisions that might affect their health (direct-to-consumer tests or DTCs). As for criteria in the SOCIAL field, the top group includes "Genetic Counseling" due to the importance of medical monitoring in the diagnosis and treatment of genetic diseases. This top group includes sub-criteria with an A and D prioritization approach.

The middle group, with four to six citations, involves fourteen Sub-criteria (numbers 9 to 22), from which three belong to the LEGAL field—"Protection of the Information," "Testing," and especially the "Health Regulation"—, while other three correspond to the SOCIAL field—"Training," "Reporting of Results" and "Accessibility.” Therefore, middle group sub-criteria include B, C and D prioritization. The eight sub-criteria in the bottom group (numbers 23 to 29), turn up only between one and three times and mainly regarding the LEGAL criterion of "Commercialization" because, as pointed out previously, few documents contemplate the use and commercialization of GGT technology within ELSI studies. However, the most up-to-date review documents (MGS, GQA, IBC) have already incorporated this criterion. This bottom group is related to prioritization approach B.

In addition, Table 3 shows the criteria that we found relevant for a general prioritization approach in the three ELSI fields, corresponding to the urgency remarks pointed out by the analyzed documents from international organizations.

**Table 3:** Priority criteria for genetic/genomic testing according to the analyzed documents

Ethical	Legal	Social
Patient rights	Protection of the information and biological samples	Counseling Accessibility
Non-discrimination	Health Regulation	Training

The seven priority criteria here distinguished are vastly represented in the analyzed documents, as shown in Fig. 4. The ETHICAL priority criteria of "Patient Rights" and "Non-discrimination” (prioritization approach A), as well as the SOCIAL priority criterion of "Counseling" (prioritization approach D), appear at the top group in number of citations, and the rest of priority criteria in Table 3 occur in more than half of the documents analyzed. Moreover, the first two of such criteria, along with "Protection of the Information", have remained present from the first published documents elaborated with a purely ethical approach and their prioritization is still maintained. The "Health Regulation" criterion (prioritization approach B) was recognized as a priority about a decade after the first documents appeared, due to the growth in the use of GGT technology either for health purposes or other interests. The priorities of ensuring tests safety and the overall quality that laboratories provide to users are now widely acknowledged as well, as it occurs increasingly also with the “Accessibility” (prioritization approach C) and the “Training” criteria (prioritization approach B). We noticed progressivity in appreciation of ELSI criteria priorities, which is understandable, as their development over time responds to varying social, cultural and economic circumstances in different countries.

Our present observations for GGT are consistent with the WHO 2002 [19] deliberations on the ETHICAL and LEGAL priority criteria and the "Counseling" criterion. They pay particular attention to "Informed Consent" and protection of "Privacy and Confidentiality" to avoid discrimination. Furthermore, Granados-Moreno et al. [10] identified as especially relevant criteria for GGT "Counseling," "Validity and Clinical utility," "Confidentiality of information" and "Informed Consent", all of which are contained in and in agreement with our results. They considered also as relevant "Genetic Exceptionalism" and "Commercialization", including "Intellectual Property" and "Direct-to-consumer Testing", which seems to us a reasonable bioeconomy prospect for the rapid development of personalized medicine. WHO 2002 and Granados-Moreno et al. 2018 also identified as priority the "Reporting of results" criterion—"Communication of risks" and "Unexpected findings."—(prioritization approach D), and we certainly realize that the "Reporting of results" criterion is essential, since it appears in more than half of the documents here analyzed (Fig. 4). The prioritization approaches A, B and C, and even “Counseling” (prioritization approach D) can be supported by national legislations through laws, regulations and sanctions. Because our priority criteria are here aimed at primary wide-coverage efforts to define national GGT regulations beyond personalized GGT care, the “Reporting of results” criterion was not contemplated. Hence, it remains pending to seek synergy between health authorities, medical academies, physicians and patients to issue guidelines for the communication of results and their implications between doctor-patient, such as in the recent Declaration of Cordoba [53] and the Report of the International Bioethics Committee on the Principle of Individual Responsibility as related to Health [54].

The ELSI criteria have been progressing in step with GGT technological advancement and growing lobbying space for introducing health national laws. Although the UDHG is not an internationally binding document, several countries worldwide use it as a basis for developing their national laws. It certainly represents a good starting point; however, as already noted above, it was originally conceived to cover ethical issues in human genome research. Leaving aside other relevant and more complex aspects of ELSI studies—such as the use and commercialization of technology, the regulation of which is becoming increasingly necessary—, we firmly believe that widely accepted ELSI criteria, consistent with international bioethical requirements like those identified in the present work, could serve as a robust foundation for basing national regulations on personalized genomic medicine.

There are differences in the explicit content with which the ELSI criteria are addressed in the documents here analyzed—some of them, for example, offer an incomplete description by taking only a single document as reference—, and they would be more clearly understood if updated so as to complement each other. To do this, we propose the following: for the ETHICAL field, within the "Informed Consent" considerations, here limited to adults with full consent capacity (Table 2), specifications for people who cannot consent (by partial or null capacity to consent) to a GGT tests were also included under the OC (article 6 to 9), and expand according to APOC guidelines to obtain authorization (article 10 to 12), as well as to REI recommendations on autonomy and informed consent (see Table 5 of that document). For the LEGAL aspect: we suggest considering HGD guidelines on the collection, processing, use, and storage of genetic data and biological samples, both in research and by testing companies, including cross-border operations. As regards ensuring the quality of GGT services, we recommend supplementing the GQA principles and best practices (part I, section 2) with those of APOC on quality of genetic services and clinical utility (article 5 and 6), and IBC recommendations, particularly for DTC (issue 121). We perceived an urgency for GGT technology regulation, which would benefit from the inclusion of parameters on the attribution of responsibilities to the different stakeholders (States and governments, scientists and regulatory bodies, media and educators, economic actors and for-profit companies) as mentioned by IBC (page 3 and 4), and the coverage of damage repair pointed out in UDHG (article 8). The most comprehensive recommended reasons to applying GGT tests are found in APOC for benefit of patients or family members or for biological materials and deceased persons (articles 8, 13, 14 and 15), and may be supported by those found in IBC on the understanding of illness and health (section II.1.3). For the SOCIAL aspect: we propound taking the broad criteria of genetic counseling from REI about counseling competent adults, children and adolescents, persons with diminished mental capacity, adults who abdicate moral autonomy (part II section 2); in conjunction with DR recommendations for medical students and physicians giving counseling (page 2 and 3). To address unexpected findings, we found appropriate the guidelines presented in DR due to the explicit information that the patient should receive (page 2). To support the distributive justice, the recommendations for encouraging the formation of civilian organizations might be considered according to MGS (section 4.6.5) and REI (part I 3.5). Likewise, there is an intense call to promote bioethics education of both patients and society, according to the observations described in MGS for supporting the effectiveness of genetic services and help to prevent discrimination and stigmatization (section. 4.7); along with the vision of REI on education as the key to ethical genetics services (part I section 3), and finally with GQA principles for education and training standards for laboratory personnel (part I 2.E).

## Synopsis and Perspectives

As shown above, the ethical, legal and social implications of research in the human genome constitute today a dynamic, progressive and profusely interconnected area of study. This is reflected in the evolution of published international documents and the parallel emergence of national ELSI programs. Three main attention areas stand out: (a) clinical care and doctor-patient relationship, (b) protection of rights for patients and relatives, and (c) advantages of interdisciplinary approach. Together they contribute to strengthen respect for human dignity, rights and privacy, in order to avoid discrimination for genetic reasons. The weakest points for effectively enforcing these demands lie in national health regulations and, above all, those related to commercialization of GGT technology.

As a necessary pending step to meet such challenges, here we present a first unified extract of the parameters that define each ELSI field and thus give actual meaning to the ethical, legal, and social issues in the major documents published on these subjects by international organizations in the last 30 years. Future elements can now be added to this disambiguated matrix in an orderly and organized manner, as needed to keep abreast with further developments in each field of study. In its present version it comprises the three fundamental ELSI fields relevant for genomic medicine, already customized for GGT: (1) applicable bioethical principles, (2) legal aspects in a flexible framework to fit the evolution of new technologies, and (3) the socio-cultural context of science and technology. It includes twenty-nine ELSI sub-criteria pertinent to GGT grouped into ten main criteria, along with seven top priority criteria we found to provide a precise starting point for reviewing, evaluating or proposing national regulations for GGT, in an integrative way for human rights protection and the development of bioeconomy.

The minimum criteria here identified are focused on GGT for medical purposes, but they can be easily extrapolated to other genomic medicine areas like biobanks [55], epigenetics [56], human genetic databases [57] and germline genetic modification [58]. It will undoubtedly be of interest extending this study to genetic testing of newborns and for non-medical purposes such as exploration of personal ancestry, as well as to groups of people in situations of vulnerability and with only partial capacity of consent like prisoners and mentally impaired persons. In addition, there are also criteria that become relevant in the international relations and research contexts, like "Genomic Sovereignty" [59, 60], "Data Sharing" [61, 62] and "International Cooperation" [63, 64].

Given the multidisciplinary and dynamic nature of the ELSI fields, the incorporation of other complementary fields of study is to be expected. The Organization for Economic Co-operation and Development (OECD) recognizes that the main health challenges which countries and societies will face over the coming decades can be alleviated through the invention, development and use of products and processes of biological materials, a collection of factors designated as Bioeconomy [65]. And this assortment will certainly include legal and commercial aspects such as proof of innovation, registration of intellectual property, verification of functionality and safety, and authorization for sale and distribution, all of which go beyond issues relating directly to patient rights and health professional obligations. A practical example of the involvement of bioeconomy in genomic studies for society is the GE3LS program by a Canadian government organization [48], which considers even environmental and economic issues in addition to the usual ethical, legal, and social aspects. Since all of this will likely play a key role in future ELSI studies, particularly for the commercialization of GGT technology, we suggest the incorporation of a BIOECONOMY field into studies concerning genomic medicine.

On the other hand, as ELSI studies evolution transits from concerns about human rights protection to concerns about profitable applications of technology in genomic medicine, the urgency of proper harmonized regulations is evident. The harmonized ELSI criteria are useful to create, verify, and supplement national regulations, which may balance the development of bioeconomy, social equity, but above all provide the maximum protection of human rights and dignity before any economic, legal, commercial, social and research interests. However, there is still a long way to go for bringing on and pairing ELSI discussions into the field of GGT technology commercialization. Accordingly, in parallel to GGT technology fast advance, there is a considerable and pressing need for international harmonization of ELSI criteria in both content and quantity. For it is inaccurate and confusing to continue calling “ELSI criteria” to just a few somewhat arbitrarily used ones when there are at least two dozen of proper options, which can be narrowed down to concise meanings as shown in this paper. Hence, it is crucial for international and interdisciplinary panels of experts to discuss and stress the use of correct ELSI nomenclature in all contexts, for which we hope the present contribution may prove useful.

## Data Availability

Not applicable.

## Abbreviations

APOC: Additional protocol to the convention on human rights and biomedicine, concerning genetic testing for health purposes
DNA: Deoxyribonucleic acid
DR: Ethical considerations regarding the use of genetics in health care (declaration of Reykjavik)
DTC: Direct-to-consumer genetic testing
ELSI: Ethical, legal, and social implications
GA4GH: Global alliance for genomics and health
GE3LS: Genomics and its ethical, environmental, economic, legal, and social aspects
GGT: Genetic and genomic testing
GQA: Guidelines for quality assurance in molecular genetic testing
HGD: International declaration on human genetic data
IBC: Report of the international bioethics committee on updating its reflection on the human genome and human rights
MGS: Medical genetic services in developing countries. The ethical, legal, and social implications of genetic testing and screening
OC: Convention for the protection of human rights and dignity of the human being regarding the application of biology and medicine: convention on human rights and biomedicine (Oviedo convention)
OECD: Organization for economic co-operation and development
p3G2: Policy partnerships project for genomic governance
REI: Review of ethical issues in medical genetics
UDHG: Universal Declaration on the Human Genome and Human Rights
UNESCO: United Nations Educational, Scientific and Cultural Organization
WHO: World Health Organization
WMA: World Medical Association
